# Pädiatrische Notfallpatienten in den Notaufnahmen einer deutschen Metropolregion: Eine retrospektive Querschnittsstudie über einen 1-Jahres-Zeitraum

**DOI:** 10.1007/s00063-023-01064-1

**Published:** 2023-09-13

**Authors:** Alexander Althammer, Heiko Trentzsch, Stephan Prückner, Christian Gehring, Florian Hoffmann

**Affiliations:** 1 Institut für Notfallmedizin und Medizinmanagement (INM), Klinikum der LMU München, Schillerstr. 53, 80336 München, Deutschland; 2 Universitätsklinikum Augsburg, Stenglinstraße 2, 86156 Augsburg, Deutschland; 3 Kinderklinik und Kinderpoliklinik im Dr. von Haunerschen Kinderspital, LMU Klinikum München, Kinderintensiv- und Notfallmedizin, Lindwurmstr. 4, 80337 München, Deutschland

**Keywords:** Pädiatrischer Notfall, Notfallversorgung Deutschland, Kindernotaufnahmen, Bedarfsanalyse Kindernotfälle, Epidemiologie des Kindernotfalls, Pädiatrische Notfallmedizin, Querschnittsstudien, Gesundheitssystemforschung, Gesundheitsreform, Pediatric emergency medicine, Needs assessment, Epidemiology, Cross-sectional studies, Public health systems research, Health care reform, Pediatric emergency department, Emergency medicine

## Abstract

**Hintergrund:**

Bisher existiert keine detaillierte Analyse von pädiatrischen Notfällen, die in Notaufnahmen versorgt werden. Im Rahmen der Kapazitätsplanung und anstehenden Reform der Notfallversorgung werden diese Daten aber dringend benötigt.

**Methode:**

Retrospektive multizentrische Querschnittsstudie für den Zeitraum vom 01.07.2013 bis zum 01.06.2014 der pädiatrischen Fälle in den Notaufnahmen Münchens.

**Ergebnisse:**

Es wurden insgesamt 103.830 Fälle analysiert (Alter: 6,9 ± 5,4 Jahre, Jungen/Mädchen 55 %/45 %). Es konnten 85,9 % der Fälle ambulant versorgt werden, 12,4 % (9,6 pro 100.000 Kinder) wurden auf die Normal- und 1,7 % (1,0 pro 100.000 Kinder) auf die Intensivstation aufgenommen. Der real benötigte Bettenbedarf überstieg jedoch diese Richtzahlen mit absolut benötigten 4,9 Intensiv- und 35,1 Normalstationsbetten pro Tag. Es zeigten sich Belastungsspitzen an den Nachmittagen des Mittwochs und des Freitags sowie an den Wochenenden. Jeder 8. Patient, der in einer Notaufnahme als Selbstzuweiser vorgestellt wurde, wurde stationär behandelt.

**Schlussfolgerung:**

Für die Kapazitätsplanung der stationären Notfallversorgung pädiatrischer Patienten müssen mehr Betten eingeplant werden als bevölkerungsbezogen zu erwarten sind. Die Verfügbarkeit der kassenärztlichen Versorgung beeinflusst das Patientenaufkommen in den Notaufnahmen (NA). Zur Verteilung der Patienten werden Instrumente zur medizinischen Ersteinschätzung des Behandlungsbedarfs und der Behandlungsdringlichkeit benötigt. Die im Rahmen der aktuellen Reform der Notfallversorgung geplanten Kindernotfallzentren müssen personell und finanziell angemessen ausgestattet werden, um – in enger Zusammenarbeit mit der kassenärztlichen Versorgung – den zu erwartenden Versorgungsbedarf bewältigen zu können.

**Zusatzmaterial online:**

Zusätzliche Informationen sind in der Onlineversion dieses Artikels (10.1007/s00063-023-01064-1) enthalten.

## Abstract (en)

**Background:**

To date, no detailed analysis of pediatric emergencies treated in emergency departments (ED) exists. However, in the context of capacity planning and upcoming emergency care reform in Germany, these data are urgently needed.

**Methods:**

Retrospective, multicenter cross-sectional study for the period 01 July 2013 to 01 June 2014 of pediatric cases in emergency departments in Munich.

**Results:**

A total of 103,830 cases were analyzed (age: 6.9 ± 5.4 years, boys/girls 55%/45%). A total of 85.9% of cases were treated as outpatients, 12.4% (9.6 per 100,000 children) were admitted to normal and 1.7% (1.0 per 100,000 children) to intensive care. However, the real bed requirements exceeded these guideline numbers, with an absolute requirement of 4.9 ICU beds and 35.1 normal ward beds per day. Load peaks were seen on Wednesday and Friday afternoons and on weekends. Every 8th patient who presented to an ED as a self-referral was treated as an inpatient.

**Conclusion:**

Capacity planning for inpatient emergency care of pediatric patients requires planning for more beds than can be expected on a population basis. The availability of panel physician care influences patient volume in the EDs. Initial medical assessment tools for treatment need and urgency are needed to distribute patients. The pediatric emergency centers planned as part of the current reform of emergency care must be adequately staffed and financed in order to be able to handle—in close cooperation with statutory health insurance-accredited medical care—the expected demand for care.

Die Epidemiologie des pädiatrischen Notfalls in Notaufnahmen in Deutschland ist bisher kaum untersucht. Gerade im Hinblick auf das bevorstehende Reformkonzept für die Notfallversorgung ist eine Kenntnis darüber jedoch von großer Bedeutung. Valide Planungsgrößen zu Bedarfen der pädiatrischen Notaufnahmen in Abhängigkeit der Bevölkerungsgröße, Auslastungsspitzen im Tages- und Wochenverlauf und Zusammenhänge in der Versorgungsstruktur zwischen dem niedergelassenen Sektor und der Inanspruchnahme klinischer Leistungen sind dabei von zentraler Bedeutung.

## Einleitung

Die Krankenhausnotaufnahmen verzeichnen seit Jahren eine steigende Inanspruchnahme durch hilfesuchende Erwachsene, aber auch durch Kinder und Jugendliche.

In der „Vierten Stellungnahme und Empfehlung der Regierungskommission zur Reform der Notfall- und Akutversorgung in Deutschland“ werden daher eine Anpassung der Versorgungsstrukturen und eine bessere Steuerung von Patienten in die geeigneten Behandlungsebenen unter Berücksichtigung der Dringlichkeit gefordert. Für eine verbesserte Zusammenarbeit zwischen den kassenärztlichen Vereinigungen und den Notaufnahmen (NA) sollen an Kliniken für Kinder- und Jugendmedizin, die die Voraussetzungen des Moduls Notfallversorgung Kinder erfüllen, integrierte Notfallzentren für Kinder und Jugendliche (KINZ) entstehen [1].

Eine bedarfsgerechte Kapazitätsplanung sollte den Anforderungen der Versorgungsrealität gerecht werden. Das Patientenaufkommen für NA, die primär erwachsene Patienten behandeln, ist bereits untersucht [2, 3]. Überraschenderweise existieren für die Versorgung von pädiatrischen Patienten in deutschen NA keine vergleichbaren Daten.

Im Rahmen einer Studie an 524.717 Fällen aus den Münchener NA wurde das Notfallaufkommen in einer deutschen Metropolregion beschrieben und Richtzahlen für die Kapazitätsplanung errechnet [2]. Diese Arbeit unterteilte die Patienten ungeachtet des Alters nach der Art der Versorgungseinrichtung in NA für Erwachsene und NA für Kinder und Jugendliche. Damit wurden Patienten unter 18 Jahren, die nicht in rein pädiatrischen Versorgungseinrichtungen gesehen wurden, der Gruppe der Erwachsenen zugeschlagen [2].

Ziel dieser Arbeit ist es, auf der Basis der Münchener Daten eine Gesamtbeschreibung aller Patienten unter 18 Jahren unabhängig von der Versorgungseinrichtung vorzunehmen, um epidemiologisch belastbare Daten für die bedarfsgerechte Kapazitätsplanung der KINZ in bundesdeutschen Ballungsgebieten zu erhalten.

## Methode

Im Rahmen einer retrospektiven multizentrischen Querschnittsstudie wurden die Behandlungsfälle in den NA von 14 Münchner Krankenhäusern im Zeitraum vom 01.07.2013 bis zum 30.06.2014 ausgewertet. Das umfasste 4 rein pädiatrische NA an Kinderkliniken (NA Kinder) und 10 weitere Krankenhausnotaufnahmen (NA Erwachsene). Manche Häuser führten eigenständige Notfallversorgungseinrichtungen für spezielle Versorgungsangebote (z. B. Augenklinik oder HNO; NA-Spezial). Methodik und Ergebnisse sind andernorts publiziert [2].

In diese Analyse wurden alle pädiatrischen Notfälle, definiert durch das Alter bei Vorstellung von unter 18 Jahren, eingeschlossen. Diese Gruppe wird im Folgenden als „Kinder“ bezeichnet und umfasst je nach Alter die Subgruppen: Neonaten und Säuglinge (< 1. Lebensjahr), Kleinkinder (1–2 Jahre), frühe Kindheit (3–5 Jahre), späte Kindheit (6–11 Jahre) und Adoleszente (12–17 Jahre).

Die Einwohnerzahl der Stadt München betrug im Beobachtungszeitraum durchschnittlich 1.465.307 Einwohner, davon durchschnittlich 209.863 (14,3 %) Kinder.

Rettungsdiensteinsätze waren definiert als jeder Transport zu einer Krankenhausnotaufnahme mit Krankentransportwagen (KTW), Rettungstransportwagen (RTW), Notarztwagen (NAW) oder Notarzteinsatzfahrzeug (NEF). Bei Fällen, die nicht mit den Leitstellendaten verknüpft werden konnten, wurde eine Vorstellung ohne Inanspruchnahme des Rettungsdienstes angenommen (Selbstzuweiser).

Die Planungsgröße „Aufwand“ wurde anhand der codierten diagnostischen und therapeutischen Prozeduren (z. B. anhand des Operationen- und Prozedurenschlüssels [OPS] Version 2015 oder des einheitlichen Bewertungsmaßstabs [EBM] nach der Methodik für den Münchener Notfallscore) bestimmt [2]. In dem 4‑stufigen Modell steht die Stufe 1 für den geringsten und die Stufe 4 für den höchsten Aufwand.

Es wurden die Versorgungstypen „ambulant“ und „stationär“ unterschieden. Als ambulant galten alle Fälle, die nicht stationär aufgenommen wurden. Als stationär galten Fälle, die auf eine Normal- oder Intensivstation aufgenommen wurden. Diese Gruppen wurden getrennt untersucht. Die Unterscheidung der High-care-Betten in Intensiv- oder Überwachungsbett erfolgte nicht.

Zur Charakterisierung des ambulanten Patientenanteils wurden diese Fälle mit den stationären Fällen verglichen, wobei Normal- und Intensivstationsfälle zusammengefasst wurden. Für diesen Teil der Auswertung wurden Fälle aus NA-Spezial ausgeschlossen, weil die speziellen Angebote dieser Einrichtungen nur lokal angeboten werden und für die ambulante Versorgung nicht relevant erscheinen.

Die Krankenhaushauptdiagnosen war nach ICD-10 codiert und die Art der Diagnose nach ICD-10-Kapiteln kategorisiert in „Trauma“ (Kapitel „Verletzungen, Vergiftungen und bestimmte andere Folgen äußerer Ursachen“) und Non-Trauma (alle anderen Kapitel).

Das Studienprotokoll sowie das Datenschutzkonzept wurden der Ethikkommission am Klinikum der Universität München vorgelegt, die die Beratungspflicht nach Fakultätsrecht aussetzte (Projekt-Nr. 17–530 UE).

### Statistische Analyse

Die Daten wurden deskriptiv ausgewertet. Soweit nicht anders angeben handelt es sich um Absolutwerte, Mittelwerte ± Standardabweichung oder den Anteil von 100 (%).

Um das Risiko einer stationären Aufnahme gegenüber der ambulanten Behandlung in Abhängigkeit des Zubringers zu schätzen, wurde eine logistische Regression durchgeführt. Das Risiko der abhängigen Variable „Versorgungstyp“ (stationär vs. ambulant) wurde dabei durch die unabhängigen Variablen „Alter“, „Geschlecht“, „Art des Zubringers“, „Art der Notaufnahme“, „Art der Diagnose“ und der „Wochentage“ geschätzt. Zur Interpretation der Ergebnisse wurden die Odds-Ratio (OR) mit zugehörigem 95 %-Konfidenzintervall (95 %-KI) angegeben. Die statistische Auswertung wurde mit R Development Core Team (2008; „R: A language and environment for statistical computing. R Foundation for Statistical Computing“, Wien Österreich) durchgeführt.

## Ergebnisse

**Abb. 1:**
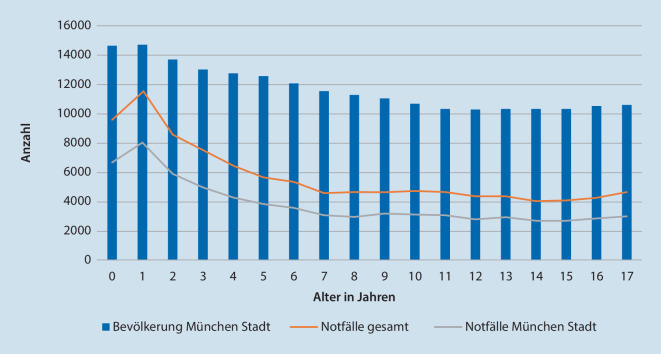
Anzahl der Bevölkerung und der Notaufnahmefälle in Abhängigkeit des Alters

Im Beobachtungszeitraum wurden 103.830 Kinder versorgt (mittleres Alter: 6,9 ± 5,4 Jahre; 55,0 % Jungen, 45 % Mädchen). Davon lebten 69.982 (67,4 %) im Stadtgebiet München. Abb. 1 stellt die Anzahl der Notfälle der jeweiligen Bevölkerungsanzahl gegenüber. Die übrigen Fälle kamen aus dem Landkreis München oder anderen geografischen Regionen.

NA-Kinder versorgten 81.185 Fälle (78,2 %); in NA-Erwachsene und NA-Spezial stellten sich 17.023 (16,4 %) bzw. 5622 (5,4 %) Fälle vor. Die Inzidenz beträgt somit 32.239 Notfallzuweisungen pro 100.000 Kinder.

Die häufigsten Diagnosegruppen nach ICD-10 für die jeweilige Altersgruppe und den Versorgungstyp sind im zusätzlichen Onlinematerial der Tabelle Z1 (Non-Trauma) und der Tabelle Z2 (Trauma) zu entnehmen.

**Abb. 2:**
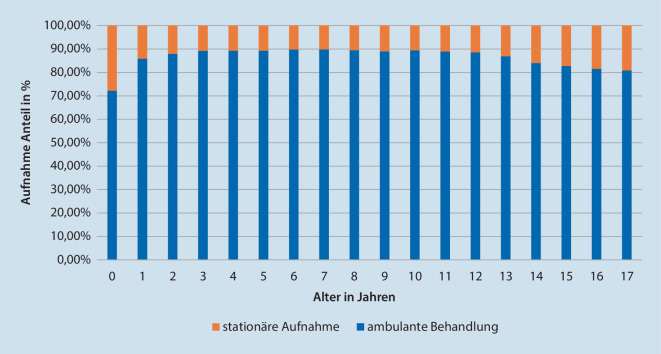
Altersabhängige Darstellung des Anteils ambulanter Fälle am Gesamtaufkommen in der Notaufnahme

Knapp 50 % der Fälle sind unter 6 Jahre alt. Die meisten Fälle konnten ambulant versorgt werden (85,9 %). Stationär wurden 12,4 % auf die Normalstation und 1,7 % auf die Intensivstation aufgenommen (Tab. 1). Relativ gesehen zeigte sich mit 5,4 % ein hoher Bedarf an intensivmedizinischen Ressourcen für die Altersgruppe Neonaten und Säuglinge (528/9731) im Gegensatz zu 1,2 % (251/20.121) für Kleinkinder, 1,2 % für frühe Kindheit (220/19661), 1,1 % für späte Kindheit (311/28.559) und 1,8 % für Adoleszente (473/25.758). Abb. 2 stellt den Anteil der ambulanten Fälle in Abhängigkeit des Patientenalters dar. Auch hier hatten Neonaten und Säuglinge einen hohen Anteil stationär versorgter Fälle.

**Tab. 1:** Aufteilung der Kinder (*n* = 103.830) auf die verschiedenen Versorgungstypen, wobei zwischen Normalstation und Intensivstation unterscheiden wurde

	Ambulant		Normalstation		Intensivstation		Gesamt	
	Anzahl	%	Anzahl	%	Anzahl	%	Anzahl	%
*Neonaten und Säuglinge*	7024	7,9	2179	17,0	528	29,6	9731	9,4
*Kleinkinder*	17.459	19,6	2411	18,8	251	14,1	20.121	19,4
*Frühe Kindheit*	17.549	19,7	1892	14,8	220	12,3	19.661	18,9
*Späte Kindheit*	25.537	28,6	2711	21,1	311	17,4	28.559	27,5
*Adoleszenz*	21.660	24,3	3625	28,3	473	26,5	25.758	24,8
*Summe*	89.229	100	12.818	100,0	1783	100,0	103.830	100,0
*Davon aus dem Stadtgebiet „München“*	61.829	–	7384	–	769	–	69.982	–

### Richtzahlen für Kapazitätsplanung

Der Gesamtbedarf von 1783 Kindern, die im einjährigen Beobachtungszeitraum über die Notaufnahmen auf Intensivstationen aufgenommen wurden, ergibt eine durchschnittliche Neuaufnahme von 4,9 Fällen pro Tag auf den Intensivstationen bzw. von 1 Neuaufnahme pro 100.000 Kinder (bezogen auf die 209.863 Kinder und vorliegenden Daten aus dem Stadtgebiet München). Die entsprechenden Werte für die Normalstationen betrugen durchschnittlich 35,1 stationäre Aufnahmen pro Tag bzw. 9,6 stationäre Fälle pro 100.000 Kinder und pro Tag. Die ambulante Versorgung in den klinischen Notaufnahmen umfasste durchschnittlich 244,5 Fälle pro Tag (80,7 pro 100.000 Kinder).

### Subanalyse und logistische Regression zur Beschreibung des hohen Anteils der ambulanten Patienten

Tab. 2 zeigt die Daten der Subanalyse und Tab. 3 zeigt die Ergebnisse der logistischen Regression.

**Tab. 2:** Charakterisierung der Fälle nach Versorgungstyp ohne Spezialkliniken

	Ambulante Behandlung	%	Stationäre Aufnahme	%	Gesamt	%
*Gesamt*	84.950	–	13.258	–	98.208	–
*Geschlecht*						
m	47.206	56,8	7139	54,4	54.345	56,4
w	35.964	43,2	5995	45,6	41.959	43,6
*Alter (Jahre)*	6,9 ± 5,2	–	7,0 ± 5,8	–	6,9 ± 5,3	–
*Aufwand*						
Gering	22.787	26,8	622	4,7	23.409	23,8
Mittel	55.675	65,5	6369	48,0	62.044	63,2
Hoch	6248	7,4	5421	40,9	11.669	11,9
Sehr hoch	240	0,3	846	6,4	1086	1,1
*Art der Notaufnahme*						
NA-Erwachsene	15.426	18,2	1597	12,0	17.023	17,3
NA-Kinder	69.524	81,8	11.661	88,0	81.185	82,7
*Zubringer*						
Selbstzuweiser	79.905	94,1	11.091	83,7	90.996	92,7
KTW	525	0,6	316	2,4	841	0,9
RTW	3464	4,1	869	6,6	4333	4,4
Notarzt	1056	1,2	982	7,4	2038	2,1
*Art der Diagnose*^a^ *(Top 3)*						
*Trauma insgesamt*	49.401	58,2	9105	68,7	58.506	59,6
Verletzungen des Kopfes	9864	61,8	2227	78,3	12.091	64,3
Verletzungen des Ellenbogens und des Unterarmes	4497	28,2	390	13,7	4887	26,0
Verletzungen der Schulter und des Oberarmes	1594	10,0	226	7,9	1820	9,7
*Non-Trauma insgesamt*	35.549	41,8	4153	31,3	39.702	40,4
Akute Infektionen der oberen Atemwege	6369	51,2	561	28,2	6930	48,1
Infektiöse Darmkrankheiten	4205	33,8	1026	51,7	5231	36,3
Symptome, die das Verdauungssystem und das Abdomen betreffen	1855	14,9	399	20,1	2254	15,6
*Wochentag*						
Montag	11.905	14,0	2084	15,7	13.989	14,2
Dienstag	10.846	12,8	1979	14,9	12.825	13,1
Mittwoch	12.228	14,4	1985	15,0	14.213	14,5
Donnerstag	11.103	13,1	1852	14,0	12.955	13,2
Freitag	12.980	15,3	2007	15,1	14.987	15,3
Samstag	13.075	15,4	1687	12,7	14.762	15,0
Sonntag	12.813	15,1	1664	12,6	14.477	14,7

^a^Detaillierte Beschreibung der Diagnosen siehe Anhang

*NA* Notaufnahme, *KTW* Krankentransportwagen, *RTW* Rettungswagen

**Tab. 3:** Risikofaktoren für eine stationäre Behandlung: Daten der logistischen Regression

Variable	Kategorie			Stationäre Aufnahme	
		Koeffizient	z‑Value	OR	95 %-KI
*Geschlecht*	m (Ref)	–	–	1	–
	w	0,1	4,4	1,1*	1,0–1,1
*Alter*	–	0,0	7,8	1,0*	1,0–1,0
	Neonaten und Säuglinge (Ref)	–	–	1	–
	Kleinkinder	−0,5	−15	0,6*	0,6–0,6
	Frühe Kindheit	−0,7	−19,3	0,5*	0,5–0,5
	Späte Kindheit	−0,7	−19,2	0,5*	0,5–0,6
	Adoleszent	−0,2	−5	0,8*	0,8–0,9
*Art der Notaufnahme*	NA-Kinder (Ref)	–	–	1	–
	NA-Erwachsene	−0,4	−12,2	0,7*	0,6–0,7
*Zubringer*	Selbstzuweiser (Ref)	–	–	1	–
	KTW	1,4	19,6	3,6*	3,1–4,1
	RTW	0,8	21,0	2,3*	2,1–2,4
	Notarzt	1,8	38,2	6,3*	5,7–6,9
*Art der Diagnose*	Non-Trauma (Ref)	–	–	1	–
	Trauma	−0,5	−26,0	0,6*	0,6–0,6
*Wochentag*	Montag (Ref)	–	–	1	–
	Dienstag	0,0	1,3	1,0	1,0–1,1
	Mittwoch	−0,1	−2,6	0,9	0,9–1,0
	Donnerstag	0,0	−1,2	1,0	0,9–1,0
	Freitag	−0,1	−3,7	0,9*	0,8–0,9
	Samstag	−0,3	−8,3	0,7*	0,7–0,8
	Sonntag	−0,3	−8,2	0,7*	0,7–0,8

Signifikanz: *p = ≤ 0,001

*Ref* Referenz, *KTW* Krankentransportwagen, *RTW* Rettungswagen

### Zubringer

92,7 % der Fälle stellen sich selbstständig in den NA vor. Der Anteil der Fälle, die mit dem Rettungsdienst kamen, macht nur 7,4 % aus, die meisten davon mit dem RTW (4,4 %). Es zeigte sich, dass das Risiko der stationären Aufnahme bei Selbstzuweisern am niedrigsten und beim Zubringer Notarzt am höchsten war (siehe Onlinematerial Abbildung Z3).

### Art der Diagnose

59,6 % der Fälle konnten der Gruppe „Trauma“ und 40,4 % der Gruppe „Non-Trauma“ zugewiesen werden. Die meisten Traumafälle kamen als Selbstzuweiser (94,3 %).

### Wochentage

Es wurden 70,2 % der Fälle unter der Woche und 29,8 % am Wochenende (Samstag und Sonntag) versorgt. Dabei war der Anteil an ambulant behandelten Fällen an Samstagen und Sonntagen mit 88,6 % bzw. 88,5 % am höchsten. An den restlichen Tagen wurden durchschnittlich 85,6 % ambulant behandelt.

**Abb. 3:**
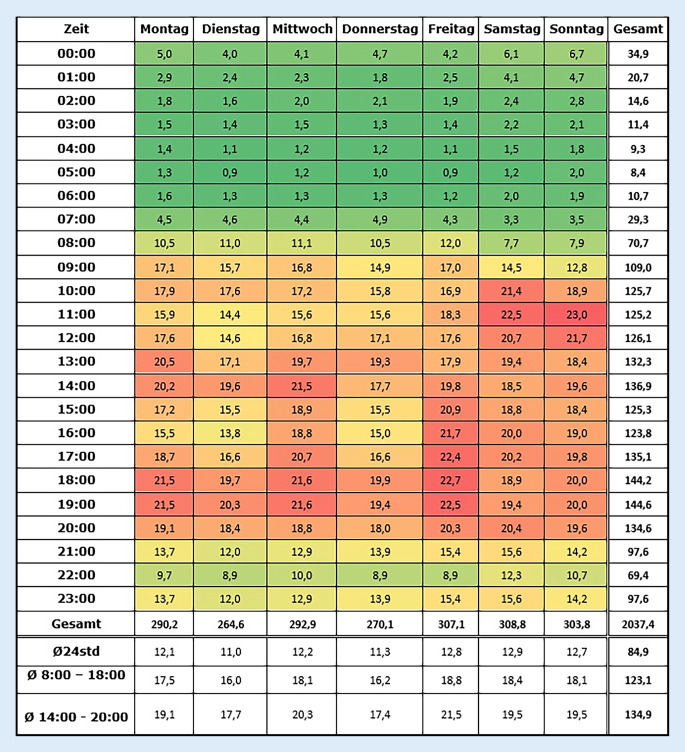
Heatmap der durchschnittlichen Anzahl der pädiatrischen Notfälle pro Tag und Stunde

Abb. 3 zeigt die durchschnittliche Anzahl an Fällen, die pro Tag und Stunde behandelt wurden. Im Durchschnitt wurden pro Stunde 12,1 Fälle behandelt. Auffällig ist das hohe Patientenaufkommen mittwochs und freitags von 14.00 Uhr bis 20.00 Uhr sowie am Wochenende.

### Stationäre Aufnahmen

Von insgesamt 90.996 Patienten, die als Selbstzuweiser die Klinik aufsuchten, wurden 11.091 stationär behandelt. Im Schnitt wurde somit jeder 8. Selbstzuweiser zur stationären Behandlung aufgenommen. Demgegenüber war das Risiko für eine stationäre Aufnahme bei Fällen, die mit dem Rettungsdienst kamen, erhöht. Beim Zubringer Notarzt zeigte sich das höchste Risiko und war 6,3-mal höher als bei Fällen, die ohne Rettungsdienst kamen. Vorstellungen am Mittwoch, Freitag sowie am Wochenende hatten ein geringeres Risiko für eine stationäre Aufnahme. Für Fälle, die in einer NA-Erwachsene behandelt wurden, sank das Risiko für eine stationäre Behandlung (OR: 0,7; 95 %-KI: 0,6–0,7). Für die Diagnoseart „Trauma“ verringerte sich das Risiko auf knapp die Hälfte.

## Limitationen

Folgenden Limitationen sind zu berücksichtigen:

- Eine zentrale Limitation dieser Arbeit stellt die fehlende Information zur Behandlungsdringlichkeit dar. Die vorhandene Triagedaten wurden mit unterschiedlichen Systemen dokumentiert und waren damit nicht vergleichbar. In den meisten Fällen fehlten Triagedaten komplett.
- Vor allem bei den ambulanten Fällen fehlten häufig Daten zu diagnostischen und therapeutischen Prozeduren, um den Behandlungsaufwand zu quantifizieren. Triage, Bett und Zubringer dienten als weitere Größen, um den Aufwand zu modellieren, weshalb die Kategorisierung nur eine sehr grobe Annäherung für den realen Behandlungsaufwand darstellt. Es lässt sich daher nicht abschätzen, ob die Behandlung der ambulanten Fälle nicht auch in der Vertragsarztpraxis hätten erbracht werden können oder ob es sich um krankenhausspezifische Leistungen handelte.
- Auch ist unbekannt, ob Selbsteinweiser vom niedergelassenen Arzt gezielt eingewiesen oder aus Eigeninitiative vorstellig wurden.
- Durch die Anonymisierung der Patientendaten ist es unmöglich zu sagen, wie viele individuelle Patienten sich vorstellten und bei wieviel Fällen es sich um Mehrfachvorstellungen handelte.

## Diskussion

Durch die flächendeckende Erfassung über ein Jahr lässt diese Studie Rückschlüsse auf die Epidemiologie des pädiatrischen Notfalls in einer deutschen Metropolregion schließen und liefert wichtige Richtzahlen für die Planung der notwendigen Behandlungskapazitäten für pädiatrische Notfallpatienten. Anders als in der Vorstudie [2] wurden hier alle Fälle unter 18 Jahre eingeschlossen. Dadurch ergibt sich ein epidemiologisch vollständiges Bild, bei dem 16,2 % der Fälle aus NA-Erwachsene und 5,4 % aus NA-Spezial eingeschlossen wurden. Die Verteilung auf ambulante und stationäre Fälle wurde dadurch allerdings kaum verändert.

Der in dieser Studie ermittelten Bedarf an Normal- und Intensivstationsbetten beträgt 9,6 bzw. 1,0 Betten/100.000 Kindern und Tag. Der tatsächlich beobachtete Bedarf lag aber mit 4,9 Intensiv- und 35,1 Normalstationsbetten pro Tag, die ausschließlich über die NA aufgenommen wurden, deutlich höher. Wahrscheinlichster Grund dafür könnte der große Einzugsbereich der Münchner Kinderkliniken sein. Wegen der insgesamt geringen Zahl von Kinderkliniken könnten solche Effekte auch in anderen Regionen zum Tragen kommen. Bedenken muss man, dass zu den Fällen aus der NA noch elektive Aufnahmen und direkte Einweisungen, die nicht über die NA laufen, hinzukommen. Für die Kalkulation von Kinderintensivbetten müssen zusätzlich innerklinische Notfälle aus dem eigenen Haus und aus peripheren Kinderkliniken, die direkt von Intensiv- zu Intensivstation verlegt wurden, berücksichtigt werden. Der reale Bettenbedarf einer Kinderklinik liegt also vermutlich deutlich höher. Ein internationaler Vergleich für die Planung der intensivmedizinischen Versorgungskapazitäten zeigt, dass der Bedarf pädiatrischer Intensivbetten aufgrund zunehmender medizinischer Komplexität zwischen 2001 und 2016 um 42 % von 5,7 pro 100.000 Kindern auf 8 pro 100.000 zugenommen hat [4].

Von besonderem Interesse für die Planung der zukünftigen Notfallversorgung pädiatrischer Patienten sind die ambulanten Fälle. Der bereits in der Voranalyse festgestellte höhere Anteil ambulanter Fälle in Kinder-NA von 85,3 % im Vergleich zu 60,2 % bei den Erwachsenen [2] war mit 86 % sogar noch geringfügig höher.

Hegenberg et al. konnten bereits zeigen, dass das Alter einen unabhängigen Risikofaktor für ambulante Behandlung bei Notaufnahmepatienten darstellt. Sie zeigten, dass ein Patientenalter kleiner 15 Jahre den stärksten Einflussfaktor für eine ambulante Behandlung ergab [5]. Für die USA wurde ein ähnlicher Zusammenhang beobachtet [6]. Mögliche Erklärungen dafür könnten eine geringe Krankheitsschwere, hoher emotionaler Druck, geringe Erfahrung im Umgang mit kranken Kindern bzw. fehlende Versorgungskapazitäten im Bereich der vertragsärztlichen Versorgung sein. Riva et al. werteten für das Jahr 2012 insgesamt 1.640.713 pädiatrische Fälle aus NA in Italien aus [7]. 59 % hiervon suchten die NA aus nichtdringlichen Gründen auf. Besonders hervorzuheben ist, dass speziell für diese Gruppe ein Zusammenhang zwischen einer schlechten niedergelassenen Versorgung und dem hohen Aufkommen in der NA gezeigt werden konnte [7]. In einer Studie von Löber et al. [8] konnte gezeigt werden, dass es sich bei mehr als der Hälfte der behandelten Patienten nach objektiver Dringlichkeitseinschätzung der Eltern nicht um einen akuten Notfall handelte. Eine Querschnittsstudie aus Deutschland zeigte, dass insbesondere junge Patienten die bessere Verfügbarkeit der NA im Vergleich zur Verfügbarkeit von niedergelassenen Ärzten als Motivation zur Inanspruchnahme der NA angeben [9, 10]. Zudem war ein besonders hoher Anteil der nichtdringlichen Patienten im Kindesalter [11].

Unsere Arbeit zeigt ein erhöhtes Patientenaufkommen in der NA im Zusammenhang zu den Öffnungszeiten der vertragsärztlichen Versorgung an den Nachmittagen des Mittwochs und des Freitags sowie an den Wochenenden. In diesen Zeiten sind Kinderarztpraxen meistens geschlossen. Am Wochenende ist das Risiko für eine stationäre Aufnahme über die NA besonders niedrig. Dies kann als Hinweis für den systemrelevanten Einfluss der niedergelassenen Ärzte auf die Auslastung der pädiatrischen NA gewertet werden. Wenn die Auslastung der NA derart von der Verfügbarkeit der kassenärztlichen Versorgung beeinflusst wird, muss man die Frage stellen, ob die Behandlungskapazitäten im niedergelassenen Bereich ausreichend bemessen sind.

Der festgestellte Aufwand war in 65,5 % mittel und in 26,8 % gering. Jedoch wurde jeder 8. Fall der Kategorie Selbstzuweiser stationär behandelt. Dies deckt sich mit Daten aus der Erwachsenenmedizin, wo bei jedem 6. Patienten eine stationäre Weiterbehandlung notwendig wurde [3], und zeigt gleichzeitig, dass in dieser Patientengruppe Fälle enthalten sind, deren Krankheitsschwere eine stationäre Aufnahme erfordert. Es scheint also nicht so zu sein, dass die ambulanten Fälle generell in den Bereich der Niedergelassenenversorgung verwiesen werden können. Um diese Fälle zeitnah und adäquat dem richtigen Versorgungssektor zuzuleiten, werden validierte Instrumente zur medizinischen Ersteinschätzung des Behandlungsbedarfs und der Behandlungsdringlichkeit benötigt, die derzeit weder für Erwachsene noch für Kinder zur Verfügung stehen und die für die Reformierung der Notfallversorgung dringend so schnell wie möglich benötigt werden. Hier besteht dringend weiterer Forschungsbedarf und die Notwendigkeit, aussagekräftige Daten bereitzustellen.

## Fazit für die Praxis

- Trotz eines hohen Anteils an ambulanten Fällen werden zur Versorgung pädiatrischer Notfälle in München mehr Betten benötigt, als dies bevölkerungsbezogen zu erwarten ist. Dies könnte am Einzugsbereich der Kliniken und an der Verfügbarkeit stationärer Behandlungseinrichtungen in der Fläche liegen.
- Wegen der vermehrten Inanspruchnahme der Notaufnahme (NA) zu Zeiten, in denen Kinderarztpraxen oft geschlossen sind, ist zu vermuten, dass die Kapazitäten der vertragsärztlichen Versorgung nicht ausreichen, um dem Bedarf gerecht zu werden.
- Perspektivisch müssen dringend Instrumente zur medizinischen Ersteinschätzung des Behandlungsbedarfs und der Behandlungsdringlichkeit für den pädiatrischen Bereich entwickelt und validiert werden, um die Patienten dem richtigen Versorgungssektor zuordnen zu können.
- Die im Rahmen der aktuellen Reform der Notfallversorgung geplanten Kindernotfallzentren müssen personell und finanziell angemessen ausgestattet werden, um – in enger Zusammenarbeit mit der kassenärztlichen Versorgung – den zu erwartenden Versorgungsbedarf bewältigen zu können.

## Supplementary Information

Tabelle Z1 (Zusatzmaterial online): Top 3 ICD-10-Kapitel und Titel der Gruppe „Non-Trauma“ nach Altersgruppen

Tabelle Z2 (Zusatzmaterial online): Top 3 ICD-10-Kapitel und Titel der Gruppe „Trauma“ nach Altersgruppen

Abbildung Z3 (Zusatzmaterial online): Stationäre Aufnahme in Prozent in Abhängigkeit der Zuweisung
