# Supplementary material for: Pediatric emergency patients in the emergency departments of a German metropolitan region: A retrospective cross-sectional study over a one-year period
Source: Med Klin Intensivmed Notfmed. 2023 Sep 13;119(6):493–501. [Article in German] doi: 10.1007/s00063-023-01064-1 (PMC11405481; doi:10.1007/s00063-023-01064-1)
Supplement: Supplementary file 1 — Tabelle Z1 (Zusatzmaterial online): Top 3 ICD-10-Kapitel und Titel der Gruppe „Non-Trauma“ nach Altersgruppen [file 63_2023_1064_MOESM1_ESM.docx]

Tabelle Z1: Top 3 ICD-10 Kapitel und Titel der Gruppe „non-Trauma“ nach Altersgruppen

|  | Anzahl | Anteil ambulant | Anteil stationär |
| --- | --- | --- | --- |
| Neonaten & Säuglinge | **7560** | **79,3%** | **20,7%** |
| Krankheiten des Atmungssystems | 1891 | 79,0% | 21,0% |
| Bestimmte infektiöse und parasitäre Krankheiten | 1311 | 80,9% | 19,1% |
| Symptome und abnorme klinische und Laborbefunde, die anderenorts nicht klassifiziert sind | 642 | 76,6% | 23,4% |
| Kleinkinder | **12850** | **85,7%** | **14,3%** |
| Krankheiten des Atmungssystems | 3879 | 80,1% | 19,9% |
| Bestimmte infektiöse und parasitäre Krankheiten | 2607 | 84,6% | 15,4% |
| Krankheiten des Ohres und des Warzenfortsatzes | 527 | 91,3% | 8,7% |
| frühe Kindheit | **11507** | **88,7%** | **11,3%** |
| Krankheiten des Atmungssystems | 2756 | 85,5% | 14,5% |
| Bestimmte infektiöse und parasitäre Krankheiten | 1996 | 85,5% | 14,5% |
| Krankheiten des Ohres und des Warzenfortsatzes | 670 | 98,2% | 1,8% |
| späte Kindheit | **13858** | **87,7%** | **12,3%** |
| Krankheiten des Atmungssystems | 1803 | 88,0% | 12,0% |
| Bestimmte infektiöse und parasitäre Krankheiten | 1774 | 86,9% | 13,1% |
| Symptome und abnorme klinische und Laborbefunde, die anderenorts nicht klassifiziert sind | 1375 | 80,8% | 19,2% |
| Adoleszent | **12731** | **78,8%** | **21,2%** |
| Symptome und abnorme klinische und Laborbefunde, die anderenorts nicht klassifiziert sind | 1812 | 71,6% | 28,4% |
| Krankheiten des Muskel-Skelett-Systems und des Bindegewebes | 1026 | 89,8% | 10,2% |
| Bestimmte infektiöse und parasitäre Krankheiten | 892 | 73,3% | 26,7% |
