# Supplementary material for: Pediatric emergency patients in the emergency departments of a German metropolitan region: A retrospective cross-sectional study over a one-year period
Source: Med Klin Intensivmed Notfmed. 2023 Sep 13;119(6):493–501. [Article in German] doi: 10.1007/s00063-023-01064-1 (PMC11405481; doi:10.1007/s00063-023-01064-1)
Supplement: Supplementary file 2 — Tabelle Z2 (Zusatzmaterial online): Top 3 ICD-10-Kapitel und Titel der Gruppe „Trauma“ nach Altersgruppen [file 63_2023_1064_MOESM2_ESM.docx]

Tabelle Z2: Top 3 ICD-10 Kapitel und Titel der Gruppe „Trauma“ nach Altersgruppen

|  | Anzahl | Anteil ambulant | Anteil stationär |
| --- | --- | --- | --- |
| Neonaten |  |  |  |
| Verletzungen, Vergiftungen und bestimmte andere Folgen äußerer Ursachen | **1122** | **72,2%** | **27,8%** |
| Verletzungen des Kopfes | 696 | 63,8% | 36,2% |
| Folgen des Eindringens eines Fremdkörpers durch eine natürliche Körperöffnung | 53 | 79,2% | 20,8% |
| Komplikationen bei chirurgischen Eingriffen und medizinischer Behandlung, anderenorts nicht klassifiziert | 36 | 69,4% | 30,6% |
| Kleinkinder |  |  |  |
| Verletzungen, Vergiftungen und bestimmte andere Folgen äußerer Ursachen | **6733** | **88,2%** | **11,8%** |
| Verletzungen des Kopfes | 3496 | 84,8% | 15,2% |
| Verbrennungen oder Verätzungen der äußeren Körperoberfläche, Lokalisation bezeichnet | 339 | 77,0% | 23,0% |
| Folgen des Eindringens eines Fremdkörpers durch eine natürliche Körperöffnung | 239 | 80,3% | 19,7% |
| frühe Kindheit |  |  |  |
| Verletzungen, Vergiftungen und bestimmte andere Folgen äußerer Ursachen | **7277** | **89,7%** | **10,3%** |
| Verletzungen des Kopfes | 3252 | 85,6% | 14,4% |
| Verletzungen des Ellenbogens und des Unterarmes | 773 | 93,1% | 6,9% |
| Verletzungen der Schulter und des Oberarmes | 421 | 88,4% | 11,6% |
| späte Kindheit |  |  |  |
| Verletzungen, Vergiftungen und bestimmte andere Folgen äußerer Ursachen | **13435** | **91,0%** | **9,0%** |
| Verletzungen des Kopfes | 3089 | 82,2% | 17,8% |
| Verletzungen des Ellenbogens und des Unterarmes | 1984 | 89,9% | 10,1% |
| Verletzungen der Schulter und des Oberarmes | 684 | 85,5% | 14,5% |
| Adoleszent |  |  |  |
| Verletzungen, Vergiftungen und bestimmte andere Folgen äußerer Ursachen | **11135** | **90,1%** | **9,9%** |
| Verletzungen des Kopfes | 1558 | 72,8% | 27,2% |
| Verletzungen des Ellenbogens und des Unterarmes | 1341 | 90,5% | 9,5% |
| Verletzungen des Knies und des Unterschenkels | 1193 | 92,3% | 7,7% |
