# Supplementary figures and images for: Pediatric emergency patients in the emergency departments of a German metropolitan region: A retrospective cross-sectional study over a one-year period
Source: Med Klin Intensivmed Notfmed. 2023 Sep 13;119(6):493–501. [Article in German] doi: 10.1007/s00063-023-01064-1 (PMC11405481; doi:10.1007/s00063-023-01064-1)

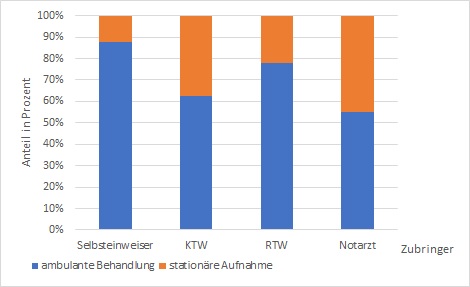

Supplement: Supplementary file 3 — Abbildung Z3 (Zusatzmaterial online): Stationäre Aufnahme in Prozent in Abhängigkeit der Zuweisung [file 63_2023_1064_MOESM3_ESM.jpg]
